# A Pattern of Antibiotic Resistance in Gram-Negative Rods Causing Urinary Tract Infection in Adults

**DOI:** 10.7759/cureus.12977

**Published:** 2021-01-28

**Authors:** Syed Asad Ali, Snigdhendu Mandal, Athanasios Georgalas, Syed Anas D Gilani

**Affiliations:** 1 Department of Medicine, Russells Hall Hospital Dudley, Dudley, GBR; 2 Department of Pharmacy/Antimicrobials Therapy, Russells Hall Hospital Dudley, Dudley, GBR

**Keywords:** gram negative rods, urinary tract infection, antibiotic resistance

## Abstract

Background and aim

Gram-negative rods (GNR) are the most common pathogens associated with urinary tract infections (UTI). The resistance of these gram-negative rods to various antibiotics is increasing with time. The study aimed to determine the pattern of resistance to antibiotics in GNR causing urinary tract infection in adults.

Material and methods

This is a cross-sectional study conducted during six months (1st December 2019 to 1st June 2020) among adult patients admitted to Russells Hall Hospital Dudley, UK. Urine cultures of 156 patients admitted with urinary tract infection were collected and reviewed. Sources of urine included midstream urine (MSU), catheter specimen urine (CSU), and others from nephrostomy bags and urine bags. Sensitivity and resistance were checked using Clinical and Laboratory Standards Institute (CLSI) guidelines. Results were analyzed using SPSS version 13.

Results

Altogether 156 patients were included in the study. Males were 40.4% of the patients were males, and 59.6% were females. The mean age was 78 with a standard deviation (SD) of 13.15. Most of the samples (67.3%) were urine MSU, 23.1% catheter urine, and 9.6% were others, like from nephrostomy bags or unspecified. Resistance to amoxicillin was found in 61.7%, trimethoprim in 36.2%, nitrofurantoin in 13.2%, ciprofloxacin in 25.6%, fosfomycin in 10.7%, co-amoxiclav in 36.2%, gentamicin in 12.8%, piperacillin-tazobactam in 7.1%, cephalexin in 44.4%, and meropenem in 0% of patients.

Conclusion

Resistance to gram-negative rods causing urinary tract infection is increasing; a particular concern is increased resistance to beta-lactams, trimethoprim, and quinolones.

## Introduction

Urinary tract infection (UTI) refers to the invasion and multiplication of microorganisms in any part of the urinary system. Urinary tract infections are the leading cause of presentation and admissions of the adult population to hospitals worldwide. UTIs account for more than 8.3 million office visits and more than one million hospital visits worldwide [1]. A UTI is usually diagnosed based on a new-onset of lower urinary tract symptoms, positive urine dipstick, or positive urine culture [2]. Urinary tract symptoms will include dysuria, new-onset nocturia, and cloudy urine to the naked eye. Urine dipstick is only considered valid in patients aged less than 65 years. A positive urine dipstick refers to the presence of leucocytes or nitrites. A urine culture will be considered positive only when having symptoms of UTI. These are considered positive when there are more than 100,000 colony forming units CFU/L of typical microorganisms seen. Asymptomatic bacteriuria is not treated as UTI except in pregnancy.

## Materials and methods

This is a cross-sectional study conducted during six months (1st December 2019 to 1st June 2020) among adult patients admitted to Russells Hall Hospital Dudley, UK. The sample size was 156 patients with a positive urine culture. The patient was included in the study if the patient had a positive urine culture with gram-negative rods, aged 18 to 96, and male or female gender. Exclusion criteria included the presence of positive urine culture in the last 30 days; the presence of ureterosigmoidostomy, suprapubic catheterization, previous urinary stricture, urethrovaginal or vesicovaginal fistula; positive urine culture with gram-positive cocci, gram-negative cocci or fungi; antibiotics not effective against gram-negative rods.

One hundred and fifty-six patients presenting to the Russells Hall Hospital, Dudley, UK, were selected according to inclusion and exclusion criteria. Data was collected as a part of the clinical audit after approval from hospital authorities and clinical supervisor. Urine samples of patients were placed in culture media for an incubation period of 24 hours at 37°C in order to isolate the uropathogen. Only gram-negative rods were considered for the collection of data. Isolated bacteria were tested against antibiotics in vitro on agar plates. Minimum inhibitory concentration (MIC) of antibiotic required to inhibit the growth of strain were found and compared to Clinical and Laboratory Standards Institute (CLSI) guidelines. Resistance patterns to antibiotics were seen, including penicillins (amoxicillin, amoxicillin/clavulanic acid, piperacillin-tazobactam), cephalosporin (cephalexin), aminoglycosides (gentamicin), quinolones (ciprofloxacin), trimethoprim, nitrofurantoin, fosfomycin, and carbapenems (meropenem). Data was collected using performa.

SPSS version 13 (SPSS Inc., Chicago, USA) was used for the analysis of data. For qualitative variables like gender, sensitivity, and resistance to antibiotics, frequency and percentage were calculated. For quantitative variables like age, mean and SD were calculated.

## Results

**Figure 1:**
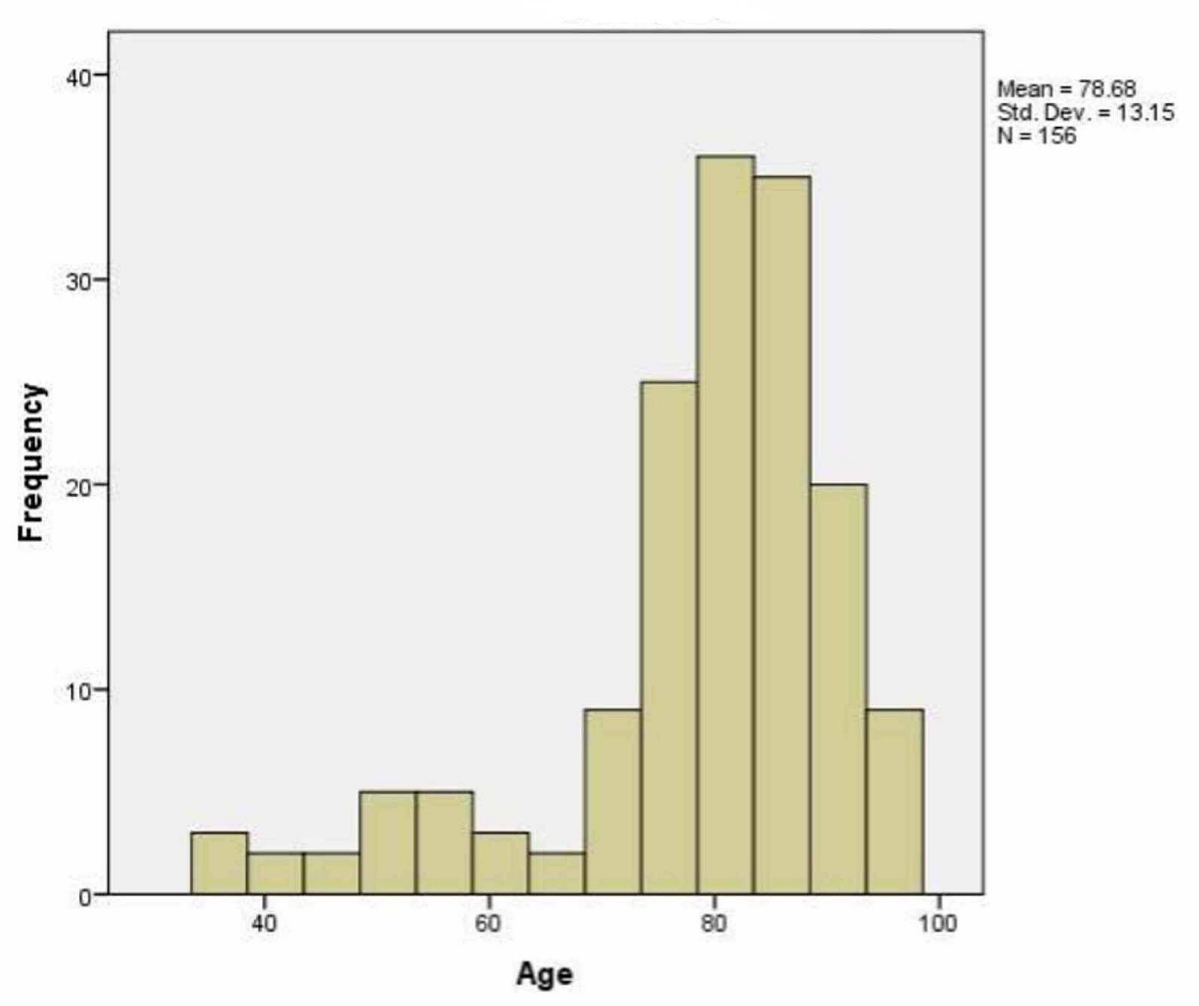
Distribution of age according to positive urine culture

A total of 156 urine cultures were analyzed. Out of 156 samples, 63 (40.4%) were from males, and 93 (59.6%) were from females. The mean age of patients admitted with gram-negative rods associated with UTI was 78 (SD=13.15, range: 36-96 years), as indicated in Figure 1. 

**Figure 2:**
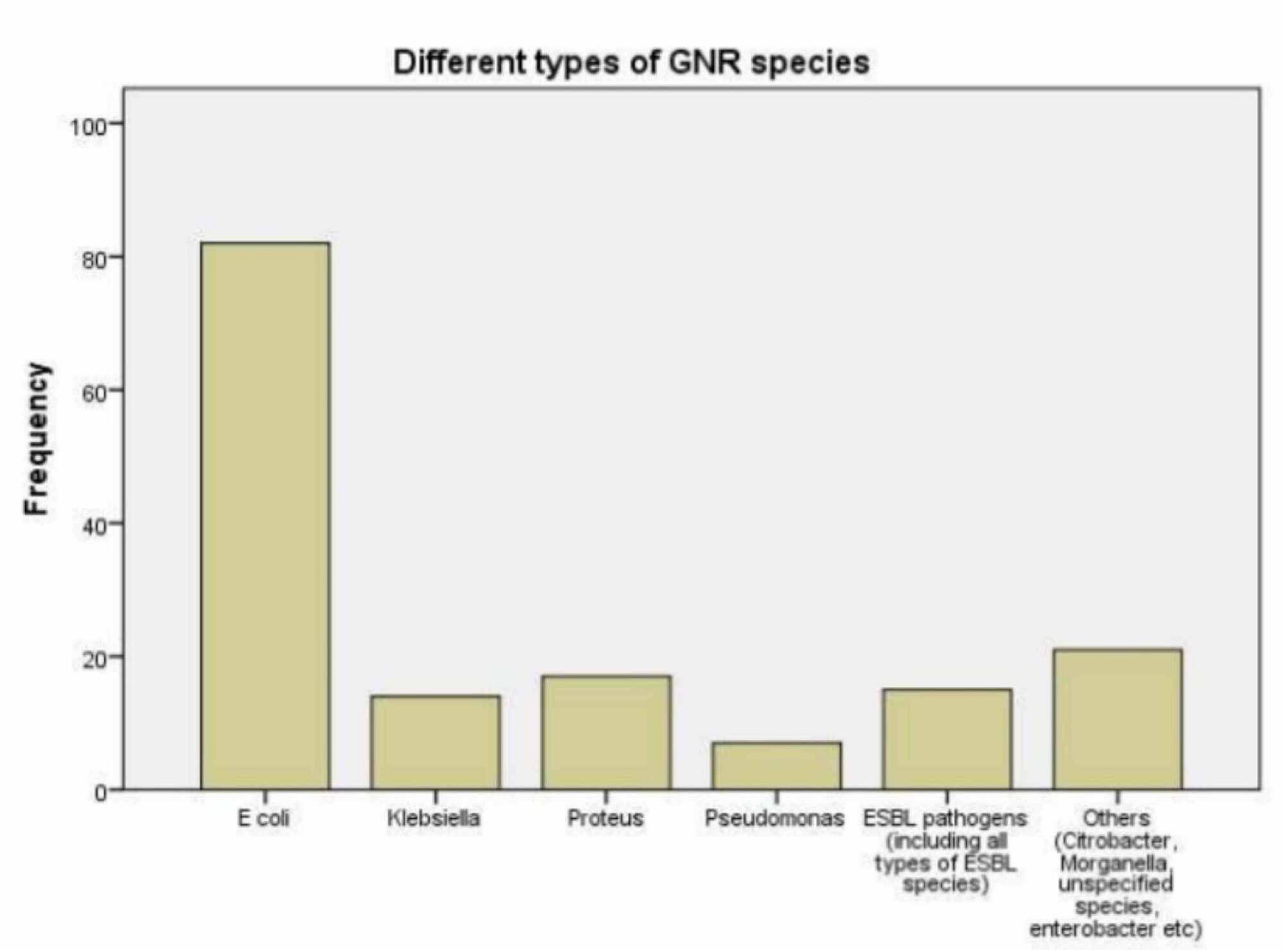
Types of gram-negative rod species isolated from urine cultures

One hundred and five samples were urine MSU (67.3%), catheter urine - 36 (23.1%), and other samples, including samples from nephrostomy, unspecified or from urine bag, were 15 (9.6%). E. coli was most commonly seen in urine cultures, 82 out of 156 samples (52.6%), Proteus in 17 (10.9%), Klebsiella in 14 (9.0%), Pseudomonas in 7 (4.5%), and other species, including Citrobacter, Morganella, Enterobacter and unspecified species were in 21 (13.5%) samples (see Figure 2). The number of ESBL pathogens isolated was 15 (9.6%). 

Amoxicillin sensitivity was checked in 149 out of 156 samples (seven samples were for Pseudomonas, for which amoxicillin is not effective). Out of 149, 92 GNR species were resistant (61.7%), and 57 sensitive (38.3%). Trimethoprim was tested for 149 samples; 93 strains were sensitive (62.4%), 54 resistant (36.2%), and two had intermediate sensitivity (1.3%). Sensitivity to nitrofurantoin was tested in 111 GNRs for Pseudomonas, Klebsiella, Enterobacter, and Morganella, which are naturally resistant to nitrofurantoin. Ninety-six strains out of 111 were sensitive (86.5%), and 15 resistant (13.5%). Ciprofloxacin showed 74.4% sensitivity (116 out of 156 ) and 25.6% resistance (40 out of 156). Sensitivity to fosfomycin was tested in 149 samples; 133 samples were sensitive (89.3%) and 16 resistant (10.7%). Co-amoxiclav was tested in 149 GNRs; 95 were sensitive (63.8%) and 54 resistant (36.2%). Gentamicin had an overall 84% sensitivity (131 out of 156), 12.8% resistance (20 out of 156), and five strains (3.2%) showed intermediate sensitivity. One hundred and thirty-five samples showed sensitivity to piperacillin-tazobactam (86.5%), 11 were resistant (7.1%), and 10 were intermediate (6.4%). Sensitivity to meropenem was tested in 152 samples, and all the strains (152) were found sensitive (100%). Sensitivity to cephalexin was checked in 90 samples, 50 were found sensitive (55.6%), and 40 were resistant (44.4%).

In summary, the resistance to antbiotics in descending order is as follows: amoxcillin 61.7%, cephalexin 44.4%, co-amoxiclav 36.2%, trimethoprim 36.2%, ciprofloxacin 25.6%, nitrofurantoin 13.5%, gentamicin 12.8%, fosfomycin 10.7%, piperacillin-tazobactam 7.1% and meropenem 0%.

## Discussion

Our study has shown that the adult population most affected by UTI is the elderly population with a mean age of 78 with SD of 13. As this study was conducted in admitted patients only, the data will tend to miss the younger patients who usually get UTIs and are not admitted, especially in the case of sexually active males and females with UTIs. Our data matches with the figures released by UK essays, which showed the chance of getting infected by UTI in women is 20% between 60-65 years age group and 20-25% over age 80. In males, the prevalence is 3% between 65-70 years and 20% above 80 years [3]. Another study shows similar results to our study - the prevalence of asymptomatic bacteriuria in care home facilities, which usually have the elderly population, is 25-50% in women having asymptomatic bacteriuria and 15-40% in men [4]. Our study showed that UTI was more common in females (59.6%) as compared to males (40.4%). This is following general data, with some studies showing the incidence of UTI in females is in a ratio of 2:1 over 70 years [5]. Similar is the data from the National Institute for Health and Care Excellence (NICE), which showed that UTIs occur in 10% of males and 20% of females over the age of 65 [6].

In our study, 67.3% specimens were urine MSU, whereas 23.1% were catheter and 9.6% of sources included nephrostomy specimens and other unspecified sources. Although our study was not aimed to compare the incidence of UTI in catheterized and non-catheterized individuals, it has proved that the incidence of UTI is significantly higher in the catheterized individuals than in non-catheterized [7]. In our study, it was not clear whether individuals having catheter specimen have symptoms of UTI before insertion of a catheter or whether it was catheter-associated UTI, but our study did show that 23.1% of patients were catheterized. It is in agreement with the Center for Disease Control (CDC) that is showing around 15-25% of patients receive catheters during hospital stay [8]. Our study has proved that E. coli is the most common pathogen associated with UTI - around 52.6%, but the percentage in our study shows only gram-negative rods and not of all pathogens, which include gram-positive cocci, fungi, etc. In addition, there were some unidentified species mentioned as coliforms, which were classified as others. Similarly, we have categorized all the E. coli extended-spectrum beta-lactamases (ESBLs) pathogens as a different category to know the exact amount of ESBLs pathogens in our data. In comparison to other studies that showed that E. coli accounts for up to 80% of community-acquired UTI [9], the above-mentioned factors might have contributed less based on our data. Another reason for the difference in data is that UTI cases in our patients were not all community-acquired. Some were catheter induced and hospital-acquired. The causes of hospital-acquired UTI do include other resistant bugs present in hospital settings, although Ecoli still remains the most common cause [10]. Our study showed that the percentage of ESBLs pathogens was around 9.6% of all gram-negative rods causing UTI. Health Protection Agency (HPA) UK estimates showed that the incidence was around 3.5% in the UK in 2003 [11]. It can be seen that the number has significantly increased. If compared to a developing country like Pakistan, it shows that ESBLs pathogens associated with UTI are much higher in Pakistan - 33.5% of E. coli pathogens are ESBL-producing, and 15.25% of Klebsiella species are ESBLs producers [12]. The reason can be related to inappropriate prescription without a diagnosis, poor compliance to antibiotics, and relatively much easier access of the general public to antibiotics in Pakistan [13].

Our study concluded that resistance to amoxicillin is 61.7%. A study from Africa in 2016 showed that resistance to amoxicillin was more than 70% only in Ecoli strains [14]. Amoxicillin, among the first penicillin to be discovered and being in use for years, has led to an evolution in gram-negative rods, and now approximately two-third of strains are resistant to it, that is why amoxicillin is not considered as the first-line antibiotic against UTI and is used only when culture is showing sensitivity. Similar is the case with cephalexin, a first-generation cephalosporin that has better gram-positive coverage; our study showed around 44.4% resistance to cephalexin. Compared to a study done in Portugal between 2000-2009 where E. coli had 14.1% resistance to a first-generation cephalosporin (cephradine) [15], our numbers show much significant increase.

The first-line of antibiotics for lower UTI is nitrofurantoin and trimethoprim, according to NICE guidelines. The resistance to trimethoprim was 34% in the UK in 2016 [16], which according to our study, is 36.2%. Similarly, the reported resistance to nitrofurantoin was 3% in the UK in 2016, which according to our study, is 13.5%. As compared to a study in India where the resistance to nitrofurantoin was 81.82%, again shows the striking difference in antibiotic resistance pattern between developed and developing countries [17]. NICE guidelines advise Ciprofloxacin or Coamoxiclav as 1st line oral agents for upper UTI (including pyelonephritis). Our study shows that ciprofloxacin has 25.6% resistance, whereas co-amoxiclav has 36.2% resistance. Figures from the United States from 1999 to 2004 showed that quinolones resistance in E. coli was only 5% with resistance to ciprofloxacin around 18% that again shows an increase in our number as compared to the past [18]. Co-amoxiclav is one of the most commonly overused antibiotics in the UK, which has led to an increase in its resistance and increase the prevalence of clostridium difficile (C. diff) associated with it. Its resistance was documented up to 51% in 2009-2014 in some hospitals in Wales, UK, after which measures were introduced to decrease its use, which showed a significant benefit [19]. Our results have shown similarities to a study done in Germany in 2017-2019, which showed 34% of the resistance of gram-negative rods to co-amoxiclav [20].

Our study showed 12.8% resistance to gentamicin and 7.1% resistance to piperacillin-tazobactam. Gentamicin is the first-line intravenous (IV) antibiotic used in patients admitted with sepsis secondary to UTI; however, impairment in renal functions and level monitoring are the few demerits of the use of gentamicin. Gentamicin resistance again is much higher in the developing world. In India, a study done in 2012 showed that all gram-negative rods, including E. coli, showed more than 50% resistance to gentamicin [21]. This again depicts the importance of standard protocols for prescribing antibiotics and the need to remove over the counter antibiotics in developing countries. Piperacillin-tazobactam is one of the broad-spectrum antibiotics which is used in life-threatening sepsis. However, data has shown that its use has increased significantly, with some studies indicating misuse by 17% [22]. However, a study from Pakistan has shown that Klebsiella strains are 20% resistant to it, and pseudomonas has 13% resistance [23].

In our study, no uropathogen was found resistant to meropenem; however, CDC has reported around 13,100 new carbapenem-resistant Enterobacteriaceae cases in hospitalized patients in the United States alone which were 11,800 cases in 2012 [24]. In the UK, data shows that there were only three carbapenems resistant cases detected in 2003, which have now increased to 1,893 in 2015 [25]. In Pakistan, a study showed around 24.1% resistance to imipenem in anaerobes [26]. Our study has shown 10.7% resistance of gram-negative rods to fosfomycin, one of the least commonly used antibiotics for UTI. It shows better sensitivity to GNR than many IV antibiotics, which makes it a potential use for the treatment of UTI as it is available in oral preparation. A study published in 2016 showed promising results of the use of fosfomycin in the treatment of UTI caused by multi-drug resistant (MDR) GNR. It showed that E. coli had 1% resistance and Klebsiella had 19% resistance to fosfomycin [27]. A recent study in Europe showed the clinical success of about 81% using IV fosfomycin as a broad-spectrum antibiotic in intensive care unit (ICU) settings for complicated infections [28].

## Conclusions

Resistance to antibiotics is increasing. Even in the case of UTI, oral antibiotics are showing much more resistance than before. A particular concern is much higher antibiotic resistance in Asia and Africa secondary to overuse and lack of antibiotic prescription control and availability of over the counter antibiotics. Immediate steps are required to control antibiotics prescription and the use of antibiotics only and when required.
